# Correspondence about “*Is the lockdown important to prevent the COVID-19 pandemic? Effects on psychology, environment and economy-perspective*” from Vol.56

**DOI:** 10.1016/j.amsu.2021.102231

**Published:** 2021-03-24

**Authors:** Oliver C. Robinson

**Affiliations:** School of Human Sciences, University of Greenwich, London, SE10 9LS, UK

Dear Editor,

I would like to express concern over the reporting of results in the article “*Is the lockdown important to prevent the COVID-19 pandemic? Effects on psychology, environment and economy-perspective*” in the Annals of Medicine and Surgery Volume 56.

An ‘unconstrained’ correlation value between lockdown days and Covid-19 cases is reported of −0.9126. This almost perfect negative correlation does not fit with the data as presented in Figs. 1 and 2. These graphs would have to be near perfect mirror images to give a negative correlation of that value, but they show signs of positive correlation. Then a table of correlations is reported between lockdown and Covid-19 cases for differing corrected distributions – all these correlations are *positive*. These positive correlations are more likely to be correct based on Figs. 1 and 2.

It would appear that either the initially reported unconstrained negative correlation in the article is incorrectly signed as negative, or the positive correlations in Table 2 are incorrectly signed as positive. It is far more likely that the positive correlations are correct, particularly given the positive correlations shown on the y axis of Fig. 4 and the data in Figs. 2 and 3.

I would be able to establish the nature of the correlation for sure if a scattergram of country-based data points had been provided, but none has been. Fig. 3 is provided, but axes are not labelled and the figure is not described, so it is impossible to say what that graph refers to. It certainly does not refer to the data on cases and lockdown, either in raw or transformed form.

The author interprets the data on the basis of a negative correlation between lockdown days and Covid-19 cases, stating that it shows lockdowns suppress the Covid pandemic. However, that is likely to be an erroneous interpretation. A positive correlation between lockdowns and mortality appears to be the case, hence the conclusion of the article would be incorrect.

Other issues with this article include the following:

• The Covid-19 case data are raw figures rather than cases per million, so case numbers are conflated with size of country, which is not appropriate.
• No hypothesis is provided, so it is unclear whether the prediction is one tailed or two tailed, hence whether a one-tailed or two-tailed significance test was selected.
• In the section on Psychological effects, the statement “It was determined that stress (8.0%) and depression (16.0–28.0%) were psychological reactions during the COVID-19 pandemic.” is provided without a citation.
• The title implies that the article looks at effects on psychology, environment and economy, but there is no data provided that relate to these issue.

